# Generalized Pentagon Equations

**DOI:** 10.1007/s00023-024-01523-1

**Published:** 2024-12-16

**Authors:** Anton Alekseev, Florian Naef, Muze Ren

**Affiliations:** 1 Section of Mathematics, University of Geneva, Rue du Conseil-Général 7-9, 1205 Geneva, Switzerland; 2 School of Mathematics, Trinity College, Dublin 2, Ireland

## Abstract

Drinfeld defined the Knizhnik–Zamolodchikov (KZ) associator $\Phi _{\textrm{KZ}}$ by considering the regularized holonomy of the KZ connection along the *droit chemin* [0, 1]. The KZ associator is a group-like element of the free associative algebra with two generators, and it satisfies the pentagon equation. In this paper, we consider paths on ${\mathbb {C}}\backslash \{ z_1, \dots , z_n\}$ which start and end at tangential base points. These paths are not necessarily straight, and they may have a finite number of transversal self-intersections. We show that the regularized holonomy *H* of the KZ connection associated with such a path satisfies a generalization of Drinfeld’s pentagon equation. In this equation, we encounter *H*, $\Phi _{\textrm{KZ}}$, and new factors associated with self-intersections, tangential base points, and the rotation number of the path.

## Introduction

The pentagon equation was first introduced in the theory of braided monoidal categories by Mac Lane [8]. In a braided monoidal category ${\mathcal {C}}$ with tensor product $\otimes$, braiding *b* and associativity morphism *a*, one has the following commuting pentagon diagram:1and two hexagon diagrams.

The Mac Lane Coherence Theorem 1.1 (originally in [8], we cite the form in [6]), explains relation between morphisms in braided monoidal categories and elements of the braid group.

### Theorem 1.1

([6, Theorem 2.9.2]) Let $X_1,\dots ,X_n\in {\mathcal {C}}$, $P_1,P_2$ be two parenthesized products of $X_1,\dots ,X_n$ (in any order) possibly with insertions of the unit object *I*, and $f,g: P_1\rightarrow P_2$ be two isomorphisms obtained by composing braiding, associativity and unit isomorphism and their inverses possibly tensored with identity morphisms. If the underlying braids are isotopic, then $f=g$.

Let ${\mathfrak {g}}$ be a complex quadratic Lie algebra (that is, a finite-dimensional Lie algebra which possesses an invariant scalar product). Drinfeld [2, 3] turned the category $U{\mathfrak {g}}-\textrm{mod}_\hbar$ of its finite-dimensional representations into a braided monoidal category using solutions of the Knizhnik–Zamolodchikov (KZ) equations.

In more detail, the KZ connection for *n* points on the complex plane

2 $$\begin{aligned} A_{\textrm{KZ}} = \frac{1}{2\pi i} \sum _{i<j} t_{i,j} \, d\log (z_i - z_j). \end{aligned}$$

takes values in the Drinfeld–Kohno Lie algebra of infinitesimal braids ${\mathfrak {t}}_{n}$ with generators $t_{ij}=t_{ji}$ with $i,j \in \{ 1, \dots , n\}$ and the relations3for distinct indices *i*, *j*, *k*, *l*. For convenience, we also use the notation

$$\begin{aligned} t_{i,jk}:=t_{i,j}+t_{i,k} \end{aligned}$$

The braiding morphisms are given by $b_{i,j} = \exp (t_{i,j}/2)$, and the associativity morphism is defined by the regularized holonomy^1^ of the KZ equation for $n=3, z_1=0, z_3=1$ and $z=z_2$ moving on the straight segment (*droit chemin*) [0, 1]. It carries the name of the KZ associator:

$$\begin{aligned} \Phi _{\textrm{KZ}}(t_{12}, t_{23}) =\textrm{Hol}^{\textrm{reg}}\left( \frac{t_{12}}{2\pi i} \, d\log (z) + \frac{t_{23}}{2\pi i} \, d\log (z-1), [0,1]\right) . \end{aligned}$$

The KZ associator satisfies the pentagon equation in $U({\mathfrak {t}}_4)$:

4 $$\begin{aligned} \Phi _{\mathrm{{KZ}}}(t_{23}, t_{34})\Phi _{\mathrm{{KZ}}}(t_{1,23}, t_{23,4})\Phi _{\mathrm{{KZ}}}(t_{12}, t_{23})=\Phi _{\mathrm{{KZ}}}(t_{12}, t_{2,34})\Phi _{\mathrm{{KZ}}}(t_{12,3}, t_{3,4}).\nonumber \\ \end{aligned}$$

In this paper, we generalize equation (4) to the case of an arbitrary number of marked points on the complex plane, and to paths which are not necessarily straight and which may have self-intersections. More precisely, we will consider the KZ equation on $\Sigma ={\mathbb {C}}\backslash \{ z_1, \dots , z_n\}$. For a point *z* moving on $\Sigma$, we associate the KZ connection

5 $$\begin{aligned} A_{z}=\frac{1}{2\pi i}\sum _{i=1}^{n} t_{i,z} \, d\log (z-z_i) \end{aligned}$$

taking values in ${\mathfrak {t}}_{n+1}$. Consider a regular path $\gamma$ which starts at a tangential base point $(z_i, v_i)$ and ends at another (might be the same) tangential base point $(z_j, v_j)$, where $v_i, v_j$ are nonvanishing vectors at $z_i$ and $z_j$, respectively (See also [5] for tangential base points.) Denote by $\textrm{rot}(\gamma )$ the rotation number of $\gamma$ with respect to the blackboard framing. We assume that $\gamma$ has a finite number of transverse self-intersection points. To each self-intersection point, one can associate the intersection number $\varepsilon _l\in \{1,-1\}, l=1, \dots , m$ indicating the orientation of the frame formed by two tangent vectors to the path at that point.

The main object of our study is the regularized holonomy

$$\begin{aligned} H = \textrm{Hol}^{\textrm{reg}}(A_z, \gamma ) \end{aligned}$$

associated with the path $\gamma$. By adding one more point *w* to the configuration, we can introduce several versions of the holonomy *H* needed in the statement of the generalized pentagon equation

6a $$\begin{aligned}  &   H_z:=\textrm{Hol}^{\textrm{reg}}\left( \frac{t_{z,wj}}{2\pi i}\, d\log (z-z_j)+\sum _{k\ne j}\frac{t_{k,z}}{2\pi i}\, d\log (z-z_k),\gamma \right) , \end{aligned}$$

6b $$\begin{aligned}  &   H_w:=\textrm{Hol}^{\textrm{reg}}\left( \frac{t_{iz,w}}{2\pi i} \, d\log (w-z_i)+\sum _{k\ne i}\frac{t_{k,w}}{2\pi i}\, d\log (w-z_k),\gamma \right) , \end{aligned}$$

6c $$\begin{aligned}  &   H_{zw}:=\textrm{Hol}^{\textrm{reg}}\left( \sum _{k=1}^n \frac{t_{k,zw}}{2\pi i} \, d\log (z-z_l),\gamma \right) . \end{aligned}$$

Note that all these versions are obtained from *H* by various substitutions, namely if we write

$$\begin{aligned} H = H(t_{1,z}, \dots , t_{n,z}) \end{aligned}$$

we have

7a $$\begin{aligned}  &   H_z= H(t_{1,z}, \dots , t_{(j-1),z}, t_{jw,z}, t_{(j+1),z}, \dots , t_{n,z}), \end{aligned}$$

7b $$\begin{aligned}  &   H_w=H(t_{1,w}, \dots , t_{(i-1),w}, t_{iz,w}, t_{(i+1),w}, \dots , t_{n,w}), \end{aligned}$$

7c $$\begin{aligned}  &   H_{zw}=H(t_{1,zw}, \dots , t_{n,zw}). \end{aligned}$$

Equipped with this notation, we can now state (a somewhat simplified version of) the generalized pentagon equation:

### Theorem 1.2

There exist elements $C_l\in \exp ({\mathfrak {t}}_{n+2})$ for $l=1, \dots , m$ such that the following identity holds in $U({\mathfrak {t}}_{n+2})$,

8 $$\begin{aligned} \begin{array}{ll} \Phi _{\mathrm{{KZ}}}(t_{zw},t_{wj}) \left( H_{zw} |v_j/v_i|^{t_{zw}/2\pi i} e^{\text {rot}(\gamma )t_{zw}}\right) \Phi _{\mathrm{{KZ}}}(t_{iz},t_{zw}) &  \\ =H_z \left( \prod _{l=1}^{m}C_{l}^{-1}e^{-\varepsilon _l t_{zw}}C_{l}\right) H_w. \end{array} \end{aligned}$$

For the case of $n=2, z_1=0, z_2=1$ and $\gamma =[0,1]$, we recover Drinfeld’s pentagon equation (4). In the general case, equation (8) contains the holonomy *H*, the KZ associator $\Phi _{\textrm{KZ}}$, as well as new terms $|v_j/v_i|^{t_{zw}/2\pi i}$ and $e^{\text {rot}(\gamma )t_{z,w}}$ depending on the tangential base points and on the rotation number of the path $\gamma$, as well as $(C_{l}^{-1}e^{-\varepsilon _l t_{z,w}}C_{l})$ which correspond to self-intersections

$$\begin{aligned} \gamma (t_l) = \gamma (s_l), \quad 0 \le t_l < s_l \le 1 \end{aligned}$$

of $\gamma$. We obtain the following partial information on $C_{l}$.

### Theorem 1.3

(see Theorem 3.3) We have,

9 $$\begin{aligned} C_l = \textrm{Hol}^{\textrm{reg}}(A_z, \gamma _{[0,t_l]}) \textrm{Hol}^{\textrm{reg}}(A_w, \gamma _{[1,s_l]}) + O(t_{zw}) \end{aligned}$$

Note that Theorem 1.2 opens the following new perspective on the topic: one can use the full KZ connection for $n+2$ points to bring the configuration $\{ z_1, \dots , z_n\}$ to a tangential base point. Then, by Mac Lane Coherence Theorem equation (8) should reduce to some equality of braids^2^ (and eventually to a number of pentagon and hexagon constraints). This new interpretation should include combinatorial expressions for the rotation number, indices of intersection points and explicit presentations of the elements $C_l$. It may possibly be related to the work of G. Massuyeau [9] in which a similar geometric setup was considered for applications to the Goldman–Turaev Lie bialgebra. We hope to return to this interesting point of view in future work.

In a forthcoming work, we plan to apply Theorem 1.2 for computing van den Bergh double brackets of universal regularized holonomies $\textrm{Hol}^{\textrm{reg}}(A_z, \gamma )$ (with values in a free associative algebra), and of Poisson brackets of the corresponding regularized holonomies for the Lie algebra ${\mathfrak {g}}=\textrm{gl}(N, {\mathbb {C}})$.

The structure of the paper is as follows. In Sect. 2.1, we discuss local solutions of the KZ equation near regular and tangential base points. In Sect. 2.2, we define regularized holonomies, and in Sect. 2.3 we recall their basic property under composition of paths. In Sect. 3.1, we follow Drinfeld and construct local solutions of the KZ equation near tangential base points. In Sect. 3.2, we analyze solutions of KZ equations self-intersection points of the path and prove the generalized Pentagon equation. Finally in subsection 3.3, we discuss the property of the terms $C_l$ in the equation.

## Regularized Holonomy

In this section, we define regularized holonomies of the KZ equation and recall their basic properties.

### Local Solutions of the KZ Equation

Let ${\mathfrak {t}}_{n+1}$ be the Drinfeld–Kohno Lie algebra in $n+1$ strands. Its generators are $t_{i,j}$ for $i,j \in \{ 1, \dots , n, z \}$ and *z* stands for $n+1$, and the relations are given by (3). Recall that the generators $t_{i,z}$ for $i=1, \dots , n$ span a free Lie subalgebra ${\mathfrak {f}}_n$ of ${\mathfrak {t}}_{n+1}$.

#### Remark 2.1

Notice that the symbol *z* denotes both the last strand and the (coordinate of the) point moving on the surface.

For the surface $\Sigma = {\mathbb {C}}\backslash \{ z_1, \dots , z_n \}$, we consider the differential equation

10 $$\begin{aligned} d\Psi =A_z\Psi , \end{aligned}$$

where $A_z$ is the flat connection given by (5) and solutions $\Psi (z) \in U{\mathfrak {f}}_n \cong {\mathbb {C}}\langle \langle t_{1,z}, \dots , t_{n,z}\rangle \rangle$ take values in the (completion of) the universal enveloping algebra of ${\mathfrak {f}}_n$.

We will associate local solutions of the equation (10) to regular points $p \in {\mathbb {C}}\backslash \{ z_1, \dots , z_n\}$ and to tangential base points $(z_i, v_i)$, where $0 \ne v_i \in {\mathbb {C}}$ is a tangent vector at $z_i$. To a regular point *p*, we associate the unique local solution $\Psi _p(z)$ which satisfies the initial condition $\Psi _p(p)=1$.

For a tangential base point $(z_i, v_i)$, let $B_\varepsilon (z_i)$ be a small open disk of radius $\varepsilon$ around $z_i$, and let $l_i=\{ z_i - sv_i; s\in {\mathbb {R}}_{\ge 0}\}$ be a ray emanating from $z_i$ in the direction opposite to $v_i$. We denote by $D_i=B_\varepsilon (z_i)\backslash l_i$ the simply connected domain obtained by deleting the ray $l_i$ from the disk $B_\epsilon (z_i)$.

#### Lemma 2.2

For $\varepsilon$ sufficiently small, there is a unique solution $\Psi _{z_i, v_i}(z)$ of the differential equation (10) on the domain $D_i$ such that

11 $$\begin{aligned} \Psi _{z_i,v_i}(z)=f\left( \frac{z-z_i}{v_i}\right) \exp \left( \frac{t_{i,z}}{2\pi i}\log \left( \frac{z-z_i}{v_i}\right) \right) , \end{aligned}$$

where *f* is an analytic function on $B_\varepsilon (0)$ with $f(0)=1$.

#### Remark 2.3

Solutions $\Psi _{z_i, v_i}(z)$ have the following asymptotic behavior for $z \rightarrow z_i$ in the domain $D_i$:

$$\begin{aligned} \Psi _{z_i, v_i}(z) \sim _{z \rightarrow z_i}\left( 1 + O\left( \frac{z-z_i}{v_i}\right) \right) \exp \left( \frac{t_{i,z}}{2\pi i}\log \left( \frac{z-z_i}{v_i}\right) \right) . \end{aligned}$$

This can also be used as a definition of these solutions.

#### Proof

For convenience of the reader, we recall the proof. Let $w=\frac{z-z_i}{v_i}$. By plugging the expression $f(w)w^{t_{iz}/2\pi i}$ into equation (10), we get

12 $$\begin{aligned} v_i^{-1}\left( f'(w)+f(w) \frac{t_{i,z}}{2\pi i w}\right) = v_i^{-1} \frac{t_{i, z}}{2\pi i w} f(w) + \sum _{j \ne i} \frac{t_{j, z}}{2\pi i (z-z_j)} f(w). \end{aligned}$$

This yields

$$\begin{aligned} f'(w) - \frac{1}{w} \left[ \frac{t_{i, z}}{2\pi i}, f(w)\right] = g(w), \end{aligned}$$

where $g(w)=\sum _k g_k w^k$ is an analytic function near zero.

We are looking for a solution in the form $f(w)=\sum _k f_k w^k$ with $f_0=1$. Since $[t_{i,z},f_0]=[t_{i, z}, 1]=0$, the expression $\frac{1}{w} \left[ \frac{t_{i, z}}{2\pi i}, f(w)\right]$ is actually regular at $w=0$. Then, we find the coefficients $f_k$ for $k \ge 1$:

$$\begin{aligned} f_k = (k - \textrm{ad}_{t_{i,z}/2\pi i})^{-1}g_{k-1}. \end{aligned}$$

This implies that the convergence radius of the power series *f* is greater than or equal to the one of the power series *g*, and hence, *f* defines an analytic function near the origin, as required. $\square$

#### Lemma 2.4

Local solutions $\Psi _p(z)$ and $\Psi _{z_i, v_i}(z)$ are group-like elements of the (completed) universal enveloping algebra $U{\mathfrak {f}}_n$.

#### Proof

For solutions at regular points, observe that the KZ connection is linear in generators of the Lie algebra ${\mathfrak {t}}_{n+1}$. Hence, $\Delta (A_z) = A_z \otimes 1 + 1 \otimes A_z$, where $\Delta$ is the standard coproduct. This implies that $\Delta (\Psi _p)$ and $\Psi _p \otimes \Psi _p$ satisfy the same differential equation

$$\begin{aligned} d {\hat{\Psi }}(z) = (A_z \otimes 1 + 1 \otimes A_z) {\hat{\Psi }}(z) \end{aligned}$$

with initial condition ${\hat{\Psi }}(p)=1 \otimes 1$. Then, by the uniqueness property of solutions of first-order ODEs we obtain $\Delta (\Psi _p)=\Psi _p \otimes \Psi _p$, as required.

Similarly, for a local solutions $\Psi _{z_i,v_i}$ we have

13 $$\begin{aligned} \Psi _{z_i,v_i}(z)=f\left( \frac{z-z_i}{v_i}\right) \exp \left( \frac{t_{i,z}}{2\pi i}\log \left( \frac{z-z_i}{v_i}\right) \right) , \end{aligned}$$

and we observe that $\Delta (f(w))$ and $f(w)\otimes f(w)$ satisfy the same differential equation

14 $$\begin{aligned} {\hat{f}}'(w)- \frac{1}{w} \left[ \frac{t_{i, z}\otimes 1+1\otimes t_{i,z}}{2\pi i}, {\hat{f}}(w)\right] =\sum _{j \ne i} \frac{v_i(t_{j, z}\otimes 1+1\otimes t_{j, z})}{2\pi i (z-z_j)} {\hat{f}}(w). \end{aligned}$$

An argument similar to the proof of existence of solutions implies uniqueness of an analytic solution of this equation with the initial value ${\hat{f}}(0)=1$. Hence, we can conclude as above.

$$\square $$

### Holonomy Maps

Let $\gamma : [0,1] \rightarrow {\mathbb {C}}$ be a smooth path with ${\dot{\gamma }}= \tfrac{d\gamma }{dt} \ne 0$, which may start and end at regular or marked points but with $\gamma (t) \in \Sigma ={\mathbb {C}}\backslash \{ z_1, \dots , z_n\}$ for all $0<t<1$. In case when $\gamma$ starts or ends at marked points (or both), we require that near that point $\gamma (t)$ be linear in *t*:

$$\begin{aligned} \begin{array}{ll} \gamma (t) = z_i + v_i t &  \textrm{for} \,\, 0< t< \varepsilon ; \\ \gamma (t) = z_j + v_j(1-t) &  \textrm{for} \,\, 0< 1-t < \varepsilon . \end{array} \end{aligned}$$

Here $(z_i, v_i)$ is the tangential starting point of $\gamma$, $(z_j, v_j)$ is the tangential end point of $\gamma$, and $\varepsilon$ is small enough. The path $\gamma (t)$ may have a finite number of transverse self-intersection points, $\gamma (s_l) = \gamma (t_l) = a_l$ for $l=1, \dots , m$. Then, we also require that $\gamma (t)$ be a linear function near the points $s_l$ and $t_l$.

To each path $\gamma (t)$, we associate two local solutions $\Psi _p(z)$ and $\Psi _q(z)$ corresponding to its end points (regular or tangential). For regular end points, we have $p=\gamma (0), q = \gamma (1)$, and for tangential end points we have $(p=(\gamma (0),{\dot{\gamma }}(0)), q = (\gamma (1), -{\dot{\gamma }}(1))$. The solutions $\Psi _p(z)$ and $\Psi _q(z)$ can be analytically continued to a strip around the path $\gamma$. More precisely, we extend $\gamma :(0,1) \rightarrow \Sigma$ to a local diffeomorphism ${\tilde{\gamma }} :(0,1) \times (-\varepsilon , \varepsilon ) \rightarrow \Sigma$. Note that the local solution $\Psi _p(z)$ defines a flat section of the pulled back connection ${\tilde{\gamma }}^*A_z$ on a neighborhood of $(0,0) \in [0,1) \times (-\varepsilon ,\varepsilon )$, which can then be analytically continued to a solution on the entire strip. The same applies to the local solution $\Psi _q(z)$. By abuse of notation, we denote analytic continuations of local solutions by the same symbols. Note that if the path $\gamma$ has self-intersections, the self-intersection point has two pre-images in the strip $(0,1) \times (-\varepsilon , \varepsilon )$, and the values of analytically continued solutions at these points are different, in general.

#### Definition 2.5

For a path $\gamma$, the (regularized) holonomy of $A_z$ along $\gamma$ is given by:

15 $$\begin{aligned} \textrm{Hol}^{\textrm{reg}}(A_z,\gamma ):=\Psi _q^{-1}(z)\Psi _p(z) \in U{\mathfrak {f}}_n, \end{aligned}$$

where *z* is any point in the strip around $\gamma$.

When both end points of $\gamma$ are regular, this definition coincides with the standard definition of a parallel transport of a connection (on a trivial bundle). In particular, in this case the holonomy is invariant under homotopies which preserve end points of the path. Similarly, for tangential end points the regularized holonomy is invariant under homotopies which preserve $(\gamma (0), {\dot{\gamma }}(0))$ for the starting point and $(\gamma (1), -{\dot{\gamma }}(1))$ for the end point.

#### Example 2.6

Consider $\Sigma = {\mathbb {C}}\setminus \{ 0, 1 \}$, tangential base points (0, 1) and $(1, -1)$, and $\gamma (t)=t$ the straight segment (*le droit chemin*) connecting 0 to 1. We have two local solutions with asymptotic behavior

$$\begin{aligned} \Psi _{0,1}(z)\sim z^{\frac{t_{1,z}}{2\pi i}}, \hspace{8.5359pt}\Psi _{1,-1}(z)\sim (1-z)^{\frac{t_{2,z}}{2\pi i}}. \end{aligned}$$

In this case, $\textrm{Hol}^{\textrm{reg}}(A_z,[0,1])$ agrees with Drinfeld’s definition of the KZ associator

$$\begin{aligned} \Phi _{\textrm{KZ}}(t_{1,z},t_{2,z})=\Psi _{1,-1}(z)^{-1} \Psi _{0,1}(z). \end{aligned}$$

#### Lemma 2.7

(Regularized) holonomies are group-like elements of the (completed) universal enveloping algebra $U{\mathfrak {f}}_n$.

#### Proof

By Lemma 2.4, local solutions are group-like. This property is preserved by analytic continuations. Since holonomies are given by ratios of two analytically continued local solutions, they are group like as well. $\square$

### Composition of Paths and Rotation Number

Two paths $\gamma _1$ and $\gamma _2$ with end points (regular or tangential) $(p_1, q_1)$ and $(p_2, q_2)$ are called composable if $p_2 = q_1$. In that case, we can define a composition of paths $\gamma _2 \gamma _1$.

**Fig. 1:**
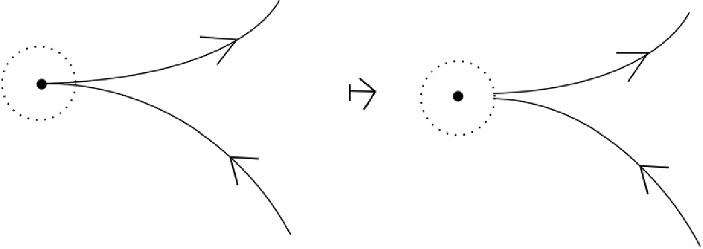
Small region near the marked point

**Fig. 2:**
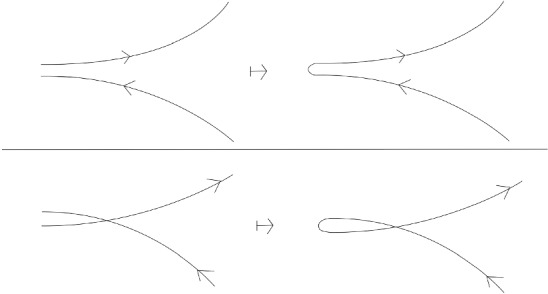
Two ways of composing curves

If the common end point $p_2 = q_1$ is a regular point, the composition is defined up to homotopy by concatenation of paths. (This allows to make the resulting path smooth.) If the common end point is a marked point, the composition is defined modulo regular homotopy (that is, the first Reidemeister move is not allowed). In that case, there are two natural ways to define the composition. One considers a neighborhood of the marked point (see Fig. 1) and adds a clockwise or a counter-clockwise half-turn with respect to the blackboard orientation of ${\mathbb {C}}$ (see Fig. 2). In this paper, we chose to use the clockwise convention.

(Regularized) holonomies are multiplicative under composition of paths:

#### Theorem 2.8

For composable paths $\gamma _1$ and $\gamma _2$, we have a product formula for the corresponding (regularized) holonomies:

16 $$\begin{aligned} \textrm{Hol}^{\textrm{reg}}(A, \gamma _2 \gamma _1) = \textrm{Hol}^{\textrm{reg}}(A, \gamma _2) \textrm{Hol}^{\textrm{reg}}(A, \gamma _1). \end{aligned}$$

#### Proof

We have

$$\begin{aligned} \textrm{Hol}^{\textrm{reg}}(A_z,\gamma _1):=\Psi _{q_1}^{-1}(z)\Psi _{p_1}(z), \hspace{8.5359pt}\textrm{Hol}^{\textrm{reg}}(A_z,\gamma _2):=\Psi _{q_2}^{-1}(z)\Psi _{p_2}(z). \end{aligned}$$

By choosing the same point *z* in both equations (*e.g.* in the small neighborhood of $\gamma _1(1) = \gamma _2(0)$), we obtain

$$\begin{aligned} \textrm{Hol}^{\textrm{reg}}(A_z,\gamma _2)\textrm{Hol}^{\textrm{reg}}(A_z,\gamma _1) = \Psi _{q_2}^{-1}(z)\Psi _{p_1}(z) = \textrm{Hol}^{\textrm{reg}}(A, \gamma _2 \gamma _1), \end{aligned}$$

as required. $$\square $$

For future use, we will need a notion of rotation number of a path. It is only defined for paths up to regular homotopy. That’s why we will be using it for paths $\gamma$ with both end points being tangential end points. Hence, we have

$$\begin{aligned} (\gamma (0), {\dot{\gamma }}(0)) = (z_i, v_i), \hspace{8.5359pt}(\gamma (1), {\dot{\gamma }}(1)) = (z_j, -v_j) \end{aligned}$$

for some marked points $z_i, z_j$ and $v_i=\rho _i e^{i \varphi _i}, v_j = \rho _j e^{i\varphi _j}$. We compute,

17 $$\begin{aligned} \int \limits _0^1 d\log ({\dot{\gamma }}(t)) = \log \ \left| \frac{v_j}{v_i} \right| + 2 \pi i \ \textrm{rot}_{\gamma }, \end{aligned}$$

where the imaginary part of this expression is the rotation number of $\gamma$. In particular, we have

$$\begin{aligned} \textrm{rot}_{\gamma } \in {\mathbb {Z}} + \frac{1}{2} + \frac{1}{2\pi }(\varphi _j - \varphi _i). \end{aligned}$$

Note that $\textrm{rot}_\gamma \in {\mathbb {R}}$ and that one full turn in the anticlockwise direction corresponding to $\textrm{rot}_\gamma =1$. For the composition of paths, we obtain

$$\begin{aligned} \textrm{rot}_{\gamma _2 \gamma _1} = \textrm{rot}_{\gamma _1} + \textrm{rot}_{\gamma _2}- \frac{1}{2}, \end{aligned}$$

where $-1/2$ on the right-hand side comes from the extra clockwise half-turn in the definition of the path composition $\gamma _2\gamma _1$.

Note that one can consistently choose $v=1$ in all tangential base points. In that case, all rotation numbers of paths are half-integers: $\textrm{rot}_\gamma \in {\mathbb {Z}} + 1/2$. Furthermore, the regular fundamental group $\pi ^{\textrm{reg}}_1(\Sigma , (z_i, v_i))$ surjects onto the ordinary fundamental group $\pi _1(\Sigma , z_i)$.

## Proof of the Generalized Pentagon Equation

In this section, we prove Theorem 1.2. We adapt Drinfeld’s original proof to the case of many marked points and to paths which may have self-intersections.

### Asymptotic Regions and Local Solutions

We consider the KZ connection for two moving points *z* and *w* in $\Sigma = {\mathbb {C}}\setminus \{ z_1, \dots , z_n \}$ with values in ${\mathfrak {t}}_{n+2}$:

18 $$\begin{aligned} A_{z,w}= \frac{1}{2\pi i}\left( \sum _{k=1}^n (t_{k,z} d\log (z-z_k) + t_{k,w}d\log (w-z_k)) + t_{z,w} d\log (z-w)\right) ,\nonumber \\ \end{aligned}$$

and the corresponding KZ equation

19 $$\begin{aligned} d\Psi = A_{z,w}\Psi . \end{aligned}$$

Let $\gamma : (0,1) \rightarrow \Sigma = {\mathbb {C}} \backslash \{ z_1, \dots , z_n\}$ be a path with a tangential starting point $(z_i, v_i)$, a tangential end point $(z_j, v_j)$, and a finite number of transverse self-intersections $a_l=\gamma (s_l) = \gamma (t_l)$ for $l=1, \dots , m$. As before, we assume that the path $\gamma$ is linear near end points and near self-intersection points.

The triangle $\{(t,s)|0\le t\le s\le 1\}\subset {\mathbb {R}}^2$ in the $t-s$ plane is mapped to the set of ordered pairs of points on $\gamma$:

$$\begin{aligned} \gamma ^{(2)}: (t,s) \mapsto (z=\gamma (t), w=\gamma (s)). \end{aligned}$$

**Fig. 3:**
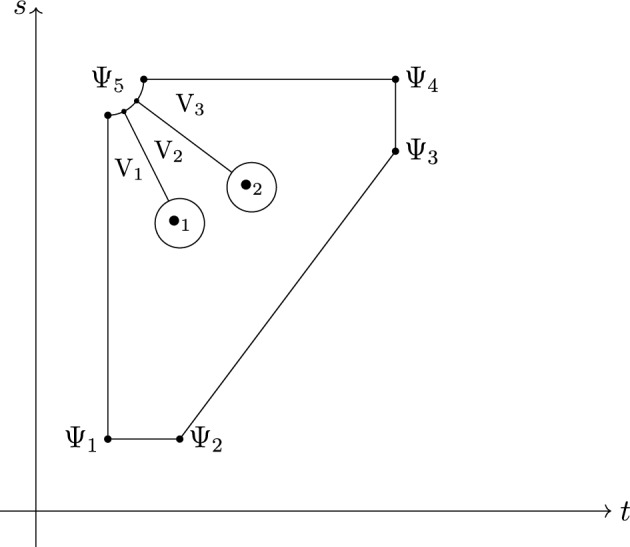
$\Psi _1,\dots ,\Psi _5$ are 5 solutions of the KZ equation and $\bullet _1,\bullet _2$ are two self-intersection points of the curve in the (t,s) plane

Following Drinfeld’s idea (see [3], and also [7]), we evaluate regularized holonomies on different parts of the closed path shown in Fig. 3. Since this path is contractible, the product of regularized holonomies is equal to 1, and this identity will give rise to the generalized pentagon equation.

In order to define regularized holonomies, we consider five asymptotic regions of the KZ equation associated with tangential base points:

20 $$\begin{aligned}&1) \hspace{8.5359pt}((z_i z) w) z_j \hspace{14.22636pt}0< t \ll s \ll 1; \nonumber \\&2) \hspace{8.5359pt}(z_i(zw))z_j \hspace{14.22636pt}0< s-t \ll s \ll 1; \nonumber \\&3) \hspace{8.5359pt}z_i ((zw)z_j) \hspace{14.22636pt}0< s-t \ll 1-t \ll 1; \nonumber \\&4) \hspace{8.5359pt}z_i (z (wz_j)) \hspace{14.22636pt}0< 1-s \ll 1-t \ll 1; \nonumber \\&5) \hspace{8.5359pt}(z_iz)(wz_j) \hspace{14.22636pt}0< t \ll 1, 0< 1-s \ll 1, \end{aligned}$$

Here parenthesized expressions of the type $((z_i z) w) z_j$ label tangential base points and the corresponding asymptotic regions.

Consider the first asymptotic region. It is convenient to introduce the new coordinates (*u*, *v*) by

$$\begin{aligned} u:=\frac{z-z_i}{w-z_i}, \quad v:=\frac{w-z_i}{v_i}. \end{aligned}$$

Let

$$\begin{aligned} D_{\varepsilon }:= \{ (u,v) \in B_{\varepsilon }(0,0) \mid u \notin {\mathbb {R}}_{<0}, v \notin v_i{\mathbb {R}}_{<0} \} \end{aligned}$$

be a small polydisc around (0, 0) in (*u*, *v*) coordinates with two branch cuts removed, so that $\log (u)$ and $\log (v)$ (taking real values on the rays opposite to branch cuts) are analytic functions on $D_\varepsilon$. Note that the first asymptotic region $0<t \ll s \ll 1$ maps to $D_\varepsilon$ by the map $\gamma ^{(2)}$. Recall the following fact:

#### Lemma 3.1

For $\varepsilon$ sufficiently small, there is a unique solution $\Psi _1$ of the KZ equation on $D_{\varepsilon }$ such that

$$\begin{aligned} \Psi _1(z,w)=f(u,v) \left( \frac{z-z_i}{v_i}\right) ^{\frac{t_{iz}}{2\pi i}}\left( \frac{w-z_i}{v_i}\right) ^{\frac{t_{iw}+t_{zw}}{2\pi i}}, \end{aligned}$$

where *f*(*u*, *v*) is an analytic function with $f(0,0)=1$.

#### Proof

See Sect. 2 in [3]. $\square$

#### Remark 3.2

We use the following shorthand notation for asymptotic behavior of the function $\Psi _1(z,w)$:

21 $$\begin{aligned} \Psi _1(z,w) \sim _{((z_iz)w)z_j}\left( \frac{z-z_i}{v_i}\right) ^{\frac{t_{iz}}{2\pi i}}\left( \frac{w-z_i}{v_i}\right) ^{\frac{t_{iw}+t_{zw}}{2\pi i}}. \end{aligned}$$

Similarly, in the other four asymptotic regions we get the following solutions:

22 $$\begin{aligned}&\Psi _2(z,w)\sim _{\left( z_i\left( z w\right) \right) z_j}\left( \frac{w-z}{v_i}\right) ^{\frac{t_{zw}}{2\pi i}} \, \left( \frac{w-z_i}{v_i}\right) ^{\frac{t_{iz}+t_{iw}}{2\pi i}}, \end{aligned}$$

23 $$\begin{aligned}&\Psi _3(z,w)\sim _{z_i\left( \left( zw\right) z_j\right) } \left( \frac{w-z}{-v_j}\right) ^{\frac{t_{zw}}{2\pi i}} \, \left( \frac{z-z_j}{v_j}\right) ^{\frac{t_{zj}+t_{wj}}{2\pi i}}, \end{aligned}$$

24 $$\begin{aligned}&\Psi _4(z,w)\sim _{z_i\left( z\left( wz_j\right) \right) } \left( \frac{w-z_j}{v_j}\right) ^{\frac{t_{wj}}{2\pi i}}\left( \frac{z-z_j}{v_j}\right) ^{\frac{t_{zw}+t_{zj}}{2\pi i}}, \end{aligned}$$

25 $$\begin{aligned}&\Psi _{5}(z,w)\sim _{(z_iz)(wz_j)}\left( \frac{z-z_i}{v_i}\right) ^{\frac{t_{iz}}{2\pi i}}\left( \frac{w-z_j}{v_j}\right) ^{\frac{t_{wj}}{2\pi i}}. \end{aligned}$$

Note that in the expressions for $\Psi _2$ and $\Psi _3$ we are using tangential base points $(0,v_i)$ and $(0, v_j)$ for the (*zw*) configuration.

### Regularized Holonomies

In this section, we compute regularized holonomies for different parts of the closed path of Fig. 3. More precisely, we extend the asymptotic solutions $\Psi _1, \dots , \Psi _4$ from above to the simply connected domain shown in Fig. 3. For $\Psi _5$, we obtain solutions

$$\begin{aligned} \Psi _{5,l}, \quad l = 1, \dots , m+1, \end{aligned}$$

where $\Psi _{5,l}$ coincides with the solution $\Psi _5$ in the region $\textrm{V}_l$. Moreover, for each intersection point $l=1,\dots ,m$ we will define two solutions $\Psi _{a_l,u},$ and $\Psi _{a_l,d}$ related by

$$\begin{aligned} \Psi _{a_l,d} = \Psi _{a_l,u} \exp (-\epsilon _l t_{z,w}). \end{aligned}$$

With that the generalized pentagon equation will follow by identifying the terms in

$$\begin{aligned}  &   \underbrace{\Psi _4^{-1} \Psi _3}_{\Phi _{\mathrm{{KZ}}} \atop \text {see (38)}} \underbrace{\Psi _3^{-1} \Psi _2}_{H_{zw} \left| \frac{v_j}{v_i} \right| ^{\frac{t_{zw}}{2\pi }} e^{t_{zw} \textrm{rot}_\gamma } \atop \text {see (36)}} \underbrace{\Psi _2^{-1} \Psi _1}_{\Phi _{\mathrm{{KZ}}} \atop \text {see (37)}}\\  &   \quad = \underbrace{\Psi _4^{-1}\Psi _{5,m+1}}_{H_z \atop \text {see (34)}} \prod _{l=1}^m \left( \underbrace{\Psi _{5,l+1}^{-1} \Psi _{a_l,u}}_{C_l^{-1}} \underbrace{\Psi _{a_l,u}^{-1} \Psi _{a_l,d}}_{e^{-\epsilon _i t_{zw}} \atop \text {see (31)}} \underbrace{\Psi _{a_l,d}^{-1} \Psi _{5,l}}_{C_l} \right) \underbrace{\Psi _{5,1}^{-1} \Psi _1}_{H_w \atop \text {see (33)}}. \end{aligned}$$

We are carrying out these identifications in the following sections. The proof of $H_{z},H_{w}, H_{zw}$ and $\Phi _{\mathrm{{KZ}}}$ contributions is similar to Sect. 2 of Drinfeld [3] and Lemma XIX.8.2 in [7]

#### Self-intersection Points

In this section, we consider solutions of (19) near self-intersection points. Suppose that the path $\gamma$ has *m* transverse self-intersection points. We order these points in the counter-clockwise direction by their positions on the $s-t$ plane, see Fig. 3. If two self-intersection points are located on the same ray, we perturb the path by a small regular homotopy. For each self-intersection point, we denote by $\varepsilon _l$ the local intersection number and by $a_l=\gamma (s_l)=\gamma (t_l)$ the position of the intersection point, where $s_l>t_l$, $l=1,\dots ,m$. By perturbing the curve $\gamma$ by a regular homotopy if needed, we can assume that $\gamma$ is a linear function for *t* near $t_l$ and $s_l$ for $l=1, \dots , m$.

We denote by $L_l$ the line in the $z-w$ plane corresponding to the straight line:

26 $$\begin{aligned} s=\frac{s_l-1}{t_l}t+1 \end{aligned}$$

in the $t-s$ plane which connects the points (0, 1) and $(t_l,s_l)$. Since $w-z=\gamma (s)-\gamma (t)$, it is convenient to introduce notation:

27 $$\begin{aligned} u_l=\frac{d}{dt} \ \left( \gamma \left( \frac{s_l-1}{t_l}t+1\right) -\gamma (t)\right) |_{t=t_l}. \end{aligned}$$

By assumptions, $\gamma (t)$ is linear in *t* near $t_l$ and $s_l$ which implies

28 $$\begin{aligned} ((z-w) \circ L_l)(t) = a_l+u_l(t-t_l),\quad \text {for}\quad |t-t_l| \quad \text {sufficiently small}. \end{aligned}$$

We denote by $B_{\varepsilon ,a_l}(z-a_l,w-z)$ the polydisk centered at (0, 0) with radius $\varepsilon$ and let $l_{a_l}=\{(z-a_l,w-z); w-z=\gamma (s)-\gamma (t),s=\frac{s_l-1}{t_l}t+1,0\le t\le t_l\}$ denote a branch cut of the function $\log (w-z)$, and we denote

$$\begin{aligned} D_{\varepsilon ,a_l}:=B_{\varepsilon ,a_l}(z-a_l,w-a_l)\setminus l_{a_l} \cap B_{\varepsilon ,a_l}(z-a_l,w-a_l). \end{aligned}$$

Similar to Theorem 4.6 in [1] (see also [4]), there is a unique solution $\Psi _{a_l, u}(z,w)$ of the KZ equation on $D_{\varepsilon ,a_l}$ such that

$$\begin{aligned} \Psi _{a_l, u}(z,w)=f(z-a_l,w-z)\exp \left( \log \left( \frac{w-z}{-u_l}\right) \frac{t_{zw}}{2\pi i} +\epsilon _l \frac{t_{zw}}{2} \right) \end{aligned}$$

where *f* is analytic on $B_{\varepsilon ,a_l}$ and $f(0,0)=1$, where

29 $$\begin{aligned} \epsilon _l = \textrm{sign} \left( \textrm{Im}\left( \frac{{\dot{\gamma }}(s_l)}{{\dot{\gamma }}(t_l)} \right) \right) \end{aligned}$$

is the sign of the intersection.

The normalization is chosen such that it satisfies the following property. If we extend $\Psi _{a_l,u}$ continuously over the line $L_l$ from above, then on the line we have

$$\begin{aligned} \Psi _{a_l,u} \sim (t-t_l)^{t_{zw}}, \end{aligned}$$

Similarly, we can define a solution $\Psi _{a_l,d}$ that has the desired asymptotics when we extend over $L_l$ from below, namely as analytic extension of

$$\begin{aligned} \Psi _{a_l, d}(z,w)=f(z-a_l,w-z)\exp \left( \log \left( \frac{w-z}{-u_l}\right) \frac{t_{zw}}{2\pi i} - \epsilon _l \frac{t_{zw}}{2} \right) , \end{aligned}$$

so that we have

$$\begin{aligned} \Psi _{a_l, d}(z,w) = \Psi _{a_l, u}(z,w) \, e^{-\varepsilon _l t_{zw}}, \end{aligned}$$

as well as

$$\begin{aligned} \lim _{(z,w) \mathrel {\downarrow }{(z_0,w_0)}} \Psi _{a_l,u}(z,w) = \lim _{(z,w) \mathrel {\uparrow }{(z_0,w_0)}} \Psi _{a_l,d}(z,w). \end{aligned}$$

where $(z_0,w_0)$ is any point on the line $L_l$ and the limits are approaching the line from above and below, respectively. Recall that $\Psi _{5,l}$ and $\Psi _{5,l+1}$ are defined by the condition that they coincide with the asymptotic solution at $(t,s)=(0,1)$ in the regions $\textrm{V}_l$ and $\textrm{V}_{l+1}$, respectively. Thus, we have

$$\begin{aligned} \lim _{(z,w) \mathrel {\downarrow }{(z_0,w_0)}} \Psi _{5,l+1}(z,w) = \lim _{(z,w) \mathrel {\uparrow }{(z_0,w_0)}} \Psi _{5,l}(z,w). \end{aligned}$$

We now define the regularized holonomy along $L_l$ to be

30 $$\begin{aligned} C_l=\textrm{Hol}^{\textrm{reg}}(A_{z,w},L_l):=\Psi _{a_l, u}^{-1} \Psi _{5, l+1}. \end{aligned}$$

From the above limit considerations, we obtain

$$\begin{aligned} C_l&= \lim _{(z,w) \mathrel {\downarrow }{(z_0,w_0)}} \Psi _{a_l, u}^{-1}(z,w) \Psi _{5, l+1}(z,w) \\&= \lim _{(z,w) \mathrel {\uparrow }{(z_0,w_0)}} \Psi _{a_l, d}^{-1}(z,w) \Psi _{5, l}(z,w) \\&= \Psi _{a_l,d}^{-1} \Psi _{5,l+1}. \end{aligned}$$

We conclude that

31 $$\begin{aligned} \Psi _{5,l+1}^{-1} \Psi _{5,l}&= \Psi _{5,l+1}^{-1} \Psi _{a_l,u} \Psi _{a_l,u}^{-1} \Psi _{a_l,d} \Psi _{a_l,d}^{-1} \Psi _{5,l} \nonumber \\&= C_l^{-1} e^{-\epsilon _l t_{zw}} C_l. \end{aligned}$$

#### $H_z$ and $H_w$

First, we consider the expression $\Psi _{5, 1}(z,w)^{-1}\Psi _1(z,w)$, where the two solutions are analytically continued in the strip of the variable *w* along the path $\gamma$.

At this point, it is convenient to introduce new functions

$$\begin{aligned} {\tilde{\Psi }}_{5,1}(z,w):=\left( \frac{z-z_i}{v_i}\right) ^{\frac{-t_{iz}}{2\pi i}}\Psi _{5,1}(z,w), \hspace{8.5359pt}{\tilde{\Psi }}_{1}(z,w):=\left( \frac{z-z_i}{v_i}\right) ^{\frac{-t_{iz}}{2\pi i}}\Psi _{1}(z,w). \end{aligned}$$

Observe that

$$\begin{aligned} \Psi _{5, 1}(z,w)^{-1}\Psi _1(z,w)={\tilde{\Psi }}_{5, 1}(z,w)^{-1} {\tilde{\Psi }}_1(z,w), \end{aligned}$$

and that functions ${\tilde{\Psi }}_{5,1}(z,w)$ and ${\tilde{\Psi }}_1(z,w)$ satisfy the same differential equation (the conjugate of the KZ equation by the factor $((z-z_i)/v_i)^{t_{iz}/2\pi i}$. Furthermore, both ${\tilde{\Psi }}_{5,1}(z,w)$ and ${\tilde{\Psi }}_1(z,w)$ are regular functions at $z=z_i$, and ${\tilde{\Psi }}_{5,1}(z_i,w)$ and ${\tilde{\Psi }}_1(z_i,w)$ are solutions of the KZ equation

32 $$\begin{aligned} d{\tilde{\Psi }}= \left( \frac{t_{iz,w}}{2\pi i} \, d\log (w-z_i)+\sum _{k\ne i}\frac{t_{k,w}}{2\pi i}\, d\log (w-z_k)\right) {\tilde{\Psi }}. \end{aligned}$$

In fact,

$$\begin{aligned} {\tilde{\Psi }}_{5,1}(z_i,w)=\Psi _{(z_j, v_j)}(w), \hspace{8.5359pt}{\tilde{\Psi }}_1(z_i,w) = \Psi _{(z_i, v_i)}(w). \end{aligned}$$

We can now conclude

33 $$\begin{aligned} \Psi _{5, 1}(z,w)^{-1}\Psi _1(z,w)&= {\tilde{\Psi }}_{5, 1}(z,w)^{-1} {\tilde{\Psi }}_1(z,w) \nonumber \\&= \Psi _{(z_j, v_j)}(w)^{-1} \Psi _{(z_i, v_i)}(w) \nonumber \\&= \textrm{Hol}^{\textrm{reg}}\left( \frac{t_{iz,w}}{2\pi i} \, d\log (w-z_i)+\sum _{k\ne i}\frac{t_{k,w}}{2\pi i}\, d\log (w-z_k),\gamma \right) \nonumber \\&= H_w. \end{aligned}$$

In a similar way, we have

34 $$\begin{aligned} \Psi _4(z,w)^{-1} \Psi _{5, m+1}(z,w)&= \textrm{Hol}^{\textrm{reg}}\left( \frac{t_{z,wj}}{2\pi i}\, d\log (z-z_j)+\sum _{k\ne j}\frac{t_{k,z}}{2\pi i}\, d\log (z-z_k),\gamma \right) \nonumber \\&= H_z. \end{aligned}$$

#### $H_{zw}$

In this section, we consider the expression $\Psi _3(z,w)^{-1} \Psi _2(z,w)$.

To start with, it is useful to consider the following simple differential equation:

$$\begin{aligned} d\phi = t_{z,w} d\log (z-w) \phi . \end{aligned}$$

We are interested in solutions of this equation for $z=\gamma (t), w=\gamma (s)$ with $t<s$. It convenient to introduce two normalized solutions $\phi _i(z,w)$ and $\phi _j(z,w)$,

35 $$\begin{aligned} \lim _{(s,t) \rightarrow (0,0)} \phi _i(z,w) \left( s-t\right) ^{-\frac{t_{zw}}{2 \pi i}} = 1, \hspace{8.5359pt}\lim _{(s,t) \rightarrow (1,1)} \phi _j(z,w) \left( s-t\right) ^{-\frac{t_{zw}}{2 \pi i}} = 1.\nonumber \\ \end{aligned}$$

The normalizations are chosen such that

$$\begin{aligned} \phi _i(z,w)&= \left( \frac{w-z}{v_i}\right) ^{\frac{t_{zw}}{2 \pi i}}, \quad \phi _j(z,w) = \left( \frac{w-z}{-v_j}\right) ^{\frac{t_{zw}}{2 \pi i}} \end{aligned}$$

in the second and third asymptotic region, respectively.

Note that expressions $\phi _i(z,w) \left( s-t\right) ^{-\frac{t_{zw}}{2 \pi i}}$ and $\phi _j(z,w) \left( s-t\right) ^{-\frac{t_{zw}}{2 \pi i}}$ admit continuous extensions to the line $s=t$, we denote these extensions by $\varphi _i(t)$ and $\varphi _j(t)$, respectively. These functions are solutions of the following differential equation:

$$\begin{aligned} d \varphi = t_{zw} \, d \log \left( \lim _{s \rightarrow t} \frac{\gamma (s) - \gamma (t)}{s-t} \right) \, \varphi = t_{zw} \, d \log {\dot{\gamma }}(t) \, \varphi . \end{aligned}$$

This implies

$$\begin{aligned} \phi _j(z, w) \phi _i^{-1}(z,w)&= \phi _j(z,w) \left( s-t\right) ^{-\frac{t_{zw}}{2 \pi i}} \left( \phi _i(z,w) \left( s-t\right) ^{-\frac{t_{zw}}{2 \pi i}} \right) ^{-1} \\&= \exp \left( t_{zw} \int \limits _ 0^1 d \log {\dot{\gamma }}(t) \right) \\&= \left| \tfrac{v_j}{v_i} \right| ^{\frac{-t_{zw}}{2\pi }} e^{-t_{zw} \textrm{rot}_\gamma }, \end{aligned}$$

where we used definition (17) in the last equation. Similarly to the previous section, we define

$$\begin{aligned} {\tilde{\Psi }}_2(z,w)=\phi _i^{-1}(z,w)\Psi _2(z,w), \hspace{8.5359pt}{\tilde{\Psi }}_3(z,w) = \phi _j^{-1}(z,w)\Psi _3(z,w). \end{aligned}$$

Again, ${\tilde{\Psi }}_2(z,w)$ and ${\tilde{\Psi }}_3(z,w)$ satisfy the same differential equation (the conjugate of the KZ equation by the factor $(w-z)^{t_{zw}/2\pi i}$), they are regular at $w=z$, and their values ${\tilde{\Psi }}_2(z=w)$ and ${\tilde{\Psi }}_3(z=w)$ satisfy the equation

$$\begin{aligned} d{\tilde{\Psi }}=\left( \sum _{k=1}^n \frac{t_{k,zw}}{2\pi i} \, d\log (z-z_l)\right) {\tilde{\Psi }}. \end{aligned}$$

By considering the asymptotic behavior, we identify

$$\begin{aligned} {\tilde{\Psi }}_2(z=w) = \Psi _{(z_i, v_i)}^{1, \dots , n, zw}(z=w), \, {\tilde{\Psi }}_3(z=w) = \Psi _{(z_j, v_j)}^{1, \dots , n, zw}(z=w). \end{aligned}$$

Using that $[t_{zw}, t_{i, zw}]=0$ for all $i=1, \dots , n$, we conclude

36 $$\begin{aligned} \Psi _3(z,w)^{-1} \Psi _2(z,w)&= {\tilde{\Psi }}_3(z=w)^{-1} \left| \frac{v_j}{v_i} \right| ^{\frac{t_{zw}}{2\pi }} e^{t_{zw} \textrm{rot}_\gamma } \, {\tilde{\Psi }}_2(z=w) \nonumber \\&= {\tilde{\Psi }}_3(z=w)^{-1} {\tilde{\Psi }}_2(z=w) \, \left| \frac{v_j}{v_i} \right| ^{\frac{t_{zw}}{2\pi }} e^{t_{zw} \textrm{rot}_\gamma } \nonumber \\&= \textrm{Hol}^{\textrm{reg}}\left( \sum _{k=1}^n \frac{t_{k,zw}}{2\pi i} \, d\log (z-z_l), \gamma \right) \left| \frac{v_j}{v_i} \right| ^{\frac{t_{zw}}{2\pi }} e^{t_{zw} \textrm{rot}_\gamma } \nonumber \\&= H_{zw} \left| \frac{v_j}{v_i} \right| ^{\frac{t_{zw}}{2\pi }} e^{t_{zw} \textrm{rot}_\gamma }. \end{aligned}$$

#### $\Phi _{\textrm{KZ}}$ Contributions

Next, we consider the regularized holonomy $\Psi _2(z,w)^{-1} \Psi _1(z,w)$. In the asymptotic region $0 < s,t \ll 1$, it is convenient to introduce

$$\begin{aligned} {\tilde{\Psi }}_{1,2}(z,w) = \left( \frac{w-z_i}{v_i} \right) ^{-(t_{iz} + t_{iw}+t_{zw})/2\pi i} \Psi _{1,2}(z,w). \end{aligned}$$

On the one hand, we have

$$\begin{aligned} {\tilde{\Psi }}_2(z,w)^{-1} {\tilde{\Psi }}_1(z,w) = \Psi _2(z,w)^{-1} \Psi _1(z,w). \end{aligned}$$

And on the other hand, $w=z_i$ is not a singularity of the differential equation, ${\tilde{\Psi }}(w=z_i)$ satisfies the following equation:

$$\begin{aligned} d {\tilde{\Psi }} = \left( t_{iz} d\log \left( \frac{z-z_i}{w-z_i} \right) + t_{zw} d\log \left( \frac{w-z}{w-z_i} \right) + \dots \right) {\tilde{\Psi }}, \end{aligned}$$

where $\dots$ stands for the terms regular in $(z-z_i), (w-z_i)$. By making the change of variables

$$\begin{aligned} \zeta = \frac{z-z_i}{w-z_i}, \hspace{8.5359pt}1-\zeta = \frac{w-z}{w-z_i} \end{aligned}$$

we identify the regularized holonomy with the Drinfeld associator

37 $$\begin{aligned} \Psi _2(z,w)^{-1} \Psi _1(z,w) = \Phi _{\textrm{KZ}}(t_{iz}, t_{zw}). \end{aligned}$$

In a similar fashion, we obtain

38 $$\begin{aligned} \Psi _4(z,w)^{-1} \Psi _3(z,w) = \Phi _{\textrm{KZ}}(t_{zw}, t_{wj}). \end{aligned}$$

### Properties of $C_l$

We now have a rather explicit description of all the terms in the generalized pentagon equation with the exception of holonomies $C_l$. In this section, we establish an important property of these regularized holonomies.

Consider the quotient of the Lie algebra ${\mathfrak {t}}_{n+2}$ by the Lie ideal generated by $t_{zw}$. Denote the quotient Lie algebra by $\tau = {\mathfrak {t}}_{n+2}/\langle t_{zw} \rangle$ and the canonical projection by $\pi : {\mathfrak {t}}_{n+2} \rightarrow \tau$. Observe that the image of generators $\pi (t_{i,z})$ and $\pi (t_{i,w})$ for $i=1, \dots , n$ span two commuting free Lie algebras with *n* generators, that is $\tau \cong {\mathfrak {f}}_z \oplus {\mathfrak {f}}_w$. Then, the image of the connection $A_{zw}$ splits into two parts:

$$\begin{aligned} \pi (A_{z,w})= &   \frac{1}{2\pi i}\left( \sum _{k=1}^n (\pi (t_{k,z}) d\log (z-z_k) + \pi (t_{k,w})d\log (w-z_k))\right) \\= &   \pi (A_z) + \pi (A_w), \end{aligned}$$

where $\pi (A_z)$ is a connection on $\Sigma = {\mathbb {C}}\backslash \{ z_1, \dots , z_n\}$ corresponding to the point *z*, and similarly $\pi (A_w)$ is a connection on $\Sigma$ corresponding to the point *w*.

#### Theorem 3.3

We have,

39 $$\begin{aligned} \pi (C_l) = \textrm{Hol}^{\textrm{reg}}(\pi (A_z), \gamma _{[0,t_l]}) \textrm{Hol}^{\textrm{reg}}(\pi (A_w), \gamma _{[1,s_l]}). \end{aligned}$$

#### Proof

Recall that

$$\begin{aligned} C_l=\Psi _{a_l, d}^{-1}(z,w)\Psi _{5, l}(z,w), \end{aligned}$$

where

$$\begin{aligned} \Psi _{a_l, d}(z,w) \sim \left( \frac{w-z}{-u_l}\right) ^{t_{zw}/2\pi i}\!, \, \Psi _{5, l}(z,w) \sim \left( \frac{z-z_i}{v_i}\right) ^{t_{iz}/2\pi i} \left( \frac{w-z_j}{v_j}\right) ^{t_{jz}/2\pi i}. \end{aligned}$$

After taking a quotient, the self-intersection point $a_l$ becomes a regular (and not tangential) base point for $\pi (A_{zw})$, and we also have

$$\begin{aligned} \pi (\Psi _{a_l, d})(a_l,a_l) = 1. \end{aligned}$$

Then,

$$\begin{aligned} \pi (\Psi _{a_l, d}) = \Psi _{a_l}(z) \Psi _{a_l}(w), \end{aligned}$$

where $\Psi _{a_l}(z)$ and $\Psi _{a_l}(w)$ are local solutions corresponding to connections $\pi (A_z)$ and $\pi (A_w)$ with the standard normalization at the regular point $\Psi _{a_l}(a_l)=1$.

Similarly, the asymptotic condition for $\Psi _{5, l}(z,w)$ splits into two independent factors:

$$\begin{aligned} \pi (\Psi _{5, l})(z,w) \sim \left( \frac{z-z_i}{v_i}\right) ^{\pi (t_{iz})/2\pi i} \left( \frac{w-z_j}{v_j}\right) ^{\pi (t_{jz})/2\pi i}, \end{aligned}$$

and hence

$$\begin{aligned} \pi (\Psi _{5, l})(z,w) = \Psi _{(z_i,v_i)}(z) \Psi _{(z_j, v_j)}(w). \end{aligned}$$

We conclude,

$$\begin{aligned} \begin{array}{lll} \pi (\Psi _{a_l, d}^{-1}(z,w)\Psi _{5, l}(z,w)) &  = &  \pi (\Psi _{a_l, d})^{-1}(z,w)\pi (\Psi _{5, l})(z,w)) \\ &  = &  (\Psi _{a_l}(z)^{-1} \Psi _{a_l}(w)^{-1})(\Psi _{(z_i,v_i)}(z) \Psi _{(z_j, v_j)}(w)) \\ &  = &  (\Psi _{a_l}(z)^{-1} \Psi _{(z_i,v_i)}(z))(\Psi _{a_l}(w)^{-1}\Psi _{(z_j, v_j)}(w)) \\ &  = &  \textrm{Hol}^{\textrm{reg}}(\pi (A_z), \gamma _{[0,t_l]}) \textrm{Hol}^{\textrm{reg}}(\pi (A_w), \gamma _{[1, s_l]}), \end{array} \end{aligned}$$

as required. $$\square $$

## Footnotes

1 See the definition in Sect. 2

2 We are grateful to D. Bar-Natan for this remark.
